# Hydroxytyrosol and Oleuropein-Enriched Extracts Obtained from Olive Oil Wastes and By-Products as Active Antioxidant Ingredients for Poly (Vinyl Alcohol)-Based Films

**DOI:** 10.3390/molecules26072104

**Published:** 2021-04-06

**Authors:** Francesca Luzi, Elisa Pannucci, Mariangela Clemente, Edoardo Grande, Silvia Urciuoli, Annalisa Romani, Luigi Torre, Debora Puglia, Roberta Bernini, Luca Santi

**Affiliations:** 1 Civil and Environmental Engineering Department, University of Perugia, Strada di Pentima 4, 05100 Terni, Italy; francesca.luzi@unipg.it (F.L.); luigi.torre@unipg.it (L.T.); 2 Department of Agriculture and Forest Sciences (DAFNE), University of Tuscia, Via San Camillo De Lellis, 01100 Viterbo, Italy; e.pannucci@unitus.it (E.P.); marian.clem@unitus.it (M.C.); edoardo.grande1986@gmail.com (E.G.); luca.santi@unitus.it (L.S.); 3 PHYTOLAB (Pharmaceutical, Cosmetic, Food Supplement, Technology and Analysis), DiSIA, University of Florence, Via Ugo Schiff 6, Sesto Fiorentino, 50019 Florence, Italy; silvia.urciuoli@gmail.com (S.U.); annalisa.romani@unifi.it (A.R.)

**Keywords:** *Olea europaea* L., hydroxytyrosol, oleuropein, agro-industrial wastes and by-products, hydroxytyrosol-enriched extract, oleuropein-enriched extract, poly (vinyl alcohol), active packaging, antioxidant activity, green chemistry, circular economy

## Abstract

Oxidative stability of food is one of the most important parameters affecting integrity and consequently nutritional properties of dietary constituents. Antioxidants are widely used to avoid deterioration during transformation, packaging, and storage of food. In this paper, novel poly (vinyl alcohol) (PVA)-based films were prepared by solvent casting method adding an hydroxytyrosol-enriched extract (HTyrE) or an oleuropein-enriched extract (OleE) in different percentages (5, 10 and 20% *w*/*w*) and a combination of both at 5% *w*/*w*. Both extracts were obtained from olive oil wastes and by-products using a sustainable process based on membrane technologies. Qualitative and quantitative analysis of each sample carried out by high performance liquid chromatography (HPLC) and nuclear resonance magnetic spectroscopy (NMR) proved that the main components were hydroxytyrosol (HTyr) and oleuropein (Ole), respectively, two well-known antioxidant bioactive compounds found in *Olea europaea* L. All novel formulations were characterized investigating their morphological, optical and antioxidant properties. The promising performances suggest a potential use in active food packaging to preserve oxidative-sensitive food products. Moreover, this research represents a valuable example of reuse and valorization of agro-industrial wastes and by-products according to the circular economy model.

## 1. Introduction

In the last few years, there has been an increasing demand for antioxidant compounds able to prevent or delay damages caused by free radicals, highly reactive and unstable species. They include reactive oxygen species (ROS) as superoxide radical (O_2_^−^·), hydroxyl radical (HO·), hydrogen peroxide (H_2_O_2_), hypochlorous acid (HClO), and reactive nitrogen species (RNS) such as nitric oxide (NO·) and peroxynitrite (ONOO^−^) [1].

Oxidative processes represent one of the main causes of food spoilage. Free radicals lead to complex and uncontrolled chain reactions that result in chemical modifications and breakdown of bioactive molecules with a consequent loss of nutritional properties and safety of food. Moreover, spoilage processes affect taste, color, texture, and shelf life of food.

Research, indeed, is involved in finding strategies to extend the shelf life of food, as often they are not consumed immediately after their production but require long transport and storage times [2].

For this reason, the addition of antioxidants to packaged food is a common practice to avoid the deleterious effects of oxidation [3]. Moreover, they can also act as dietary supplements contributing to neutralize the adverse effects of oxidative stress. Synthetic phenols as butylated hydroxyanisole (BHA) and 2,6-di-*tert*-butyl-4-methylphenol (BHT) are examples of antioxidants suitable for preservation of oils and fat-containing products. Their use has been widely recommended due the high performance, low cost and easy availability. However, in recent times, the effects of these compounds on human health are under scrutiny for their tendency to accumulate in human tissues [4].

In this respect, an innovative solution, strongly supported by both academia and industry, which aims to increase food shelf life and preserve food quality is active food packaging [5,6]. It consists in the incorporation of active ingredients (AI) into polymeric materials to achieve a controlled release over time into food.

A variety of active food packages have been produced adding pure compounds with antioxidant activities as carvacrol, thymol [7], ferulic acid, caffeic acid [8], gallic acid, quercetin [9,10], tocopherol, quercetin [11], hydroxytyrosol [12,13] as well as complex plant-derived extracts [14,15] into different polymeric films such as poly (vinyl alcohol) and poly (vinyl alcohol-co-ethylene).

Recently, an increasing attention has turned to agro-industrial wastes and by-products as source of antioxidants [16]. The recovery and reuse of these materials has the twofold advantage of reducing the input of raw materials as well as the detrimental effects on the environment due to their disposal. These features fit with to the “zero waste concept” emphasized by the circular economy model that should be maximized in each productive process [17].

In this scenario, wastes and by-products of the olive oil industry represent a very attractive plant material for obtaining polyphenols-based antioxidants useful for a variety of industrial applications [18] including design and production of active food packages.

**Figure 1:**
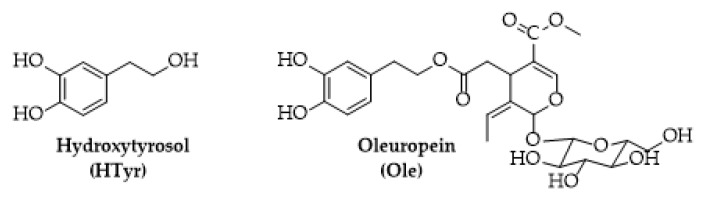
Chemical structure of hydroxytyrosol (HTyr) and oleuropein (Ole).

Hydroxytyrosol (HTyr) and oleuropein (Ole) are two bioactive constituents of olive tree (*Olea europaea* L., Figure 1) used as dietary supplements for their beneficial effects on human health or as food preservatives for their strong antioxidant activity [19]. HTyr is a low molecular weight phenol found mainly in olive fruits (16.6 mg/kg) [20]; Ole a secoiridoid can be found in both fruits (19.2 mg/kg) and leaves (26.47 mg/kg) [21].

During the production of extra virgin olive oil, a high quantity of wastes consisting of free-oil olive pulp (solid) and olive mill wastewaters (liquid) is obtained. Generally, 100 kg of extra virgin olive oil produce 400 kg of free-oil olive pulp and 100 kg of olive mill wastewaters [20]. The crushing of olive drupes releases endogenous β-glucosidases able to hydrolyze the ester bond present in Ole generating HTyr, which is partitioned between oil and water phases [22]. On the base of HTyr hydrophilic properties, this process tends to deplete HTyr from olive oil while enriching olive mill wastewaters: in fact, up to 214 mg of HTyr can be found per 1 kg of olive mill wastewaters [20]. Notably free-oil olive pulp and olive mill wastewaters represent a serious environmental problem, due to the high amount produced every year and the remarkable content in organic matter such as polyphenols, which are responsible for high toxicity [23]. For this reason, their disposal requires a significant economic effort to avoid severe environmental pollution.

Leaves are particularly rich in Ole and are indeed an olive oil industry waste mainly generated from pruning of olive trees, an agricultural practice generally carried out at the end of winter producing tons of biomass, but also at the end of autumn during olive harvesting and oil production [19].

The combination of polymeric films and AI exhibiting antioxidant activity plays a crucial and important role in areas related to human activities as the food sector. Being food packages responsible for protecting and covering most of the products consumed and distributed over the world, the industrial sector is strongly interested towards the development of active formulations.

A recent paper described the preparation and properties of novel multilayer active films based on polylactic acid/poly (butylene adipate-co-terephthalate) incorporating extracts obtained from olive oil wastewaters [24]. For their good barrier effects, surface wettability and antioxidant activity, these films have been proposed as active food packages against oxidative food deterioration. However, despite their good performances, neither the preparation of the extract nor the qualitative and quantitative content of phenolic compounds were reported in the paper and both aspects are indeed relevant for reproducibility and consequently for its proper use and application.

In this context, the membrane technologies used indeed ensure the reproducibility of the extraction process starting from the plant materials [25]; the resulting extracts can be characterized by advanced analytical techniques such as high-performance liquid chromatography (HPLC) and nuclear resonance magnetic spectroscopy (NMR) useful for their standardization [26,27].

Based on the expertise of the research group, this paper deals with the design, production, and characterization of poly (vinyl alcohol) (PVA)-based films obtained incorporating hydroxytyrosol, oleuropein-enriched extracts (HTyrE and OleE, respectively) or their combination. HTyrE and OleE were prepared from free-oil olive pulp and leaves, respectively, through membrane technologies, purified by chromatography and characterized by HPLC and NMR. Their use perfectly fits with the circular economy model.

PVA is a semi-crystalline polymer produced by hydrolysis of poly (vinyl acetate); is a water soluble, biocompatible, cheap and non-toxic film offering good thermal stability, thermomechanical characteristics, flexibility and strength, physical and optical properties relevant for packaging applications [28,29]. Furthermore, the Food and Drug Administration (FDA) officially approved PVA for producing stretchable and flexible food packaging systems [30,31]. Due to these properties, PVA was selected as the polymer matrix for the novel active packaging solutions proposed in the present research work. The choice of water casting process to obtain the novel formulations represents another eco-friendly aspect of this research, aimed to avoid the use of toxic organic solvents and heating processes.

The effect of different percentages of each extract (5, 10 and 20% *w*/*w*) and a combination of both extracts at 5% *w*/*w* on morphological behavior, optical, antioxidant and release properties of PVA was investigated, taking into account the application of these films as antioxidant materials. Finally, their antioxidant activity was evaluated at different contact times with a food simulant to test their effectiveness in active packaging.

## 2. Results and Discussion

### 2.1. Hydroxytyrosol Enriched Extract (HTyrE) and Oleuropein Enriched Extract (OleE) Preparation and Characterization

For the purposes of this work, an hydroxytyrosol-enriched extract (HTyrE) and an oleuropein-enriched extract (OleE) were prepared using, respectively, free-oil olive pulp and leaves as starting materials.

Raw materials were first subjected to a solid-liquid extraction in water using a pneumatic extractor and then filtered in sequence using different membranes (microfiltration, ultrafiltration, nanofiltration) and then reverse osmosis to obtain progressively more refined fractions at the end of each step. Fractions were then purified by chromatography to obtain the final products (HTyrE and OleE, respectively) which were characterized by HPLC-DAD and ^1^H- and ^13^C-NMR analysis.

Flash and preparative chromatography of the fractions was carried out using the Pure C-850 FlashPrep Chromatography System (Büchi Italia Srl, Milan, Italy). A C18 column was used as stationary phase, while water and ethanol as eluents. Technical and experimental details of the chromatographic process are described in Section 3.2.

**Figure 2:**
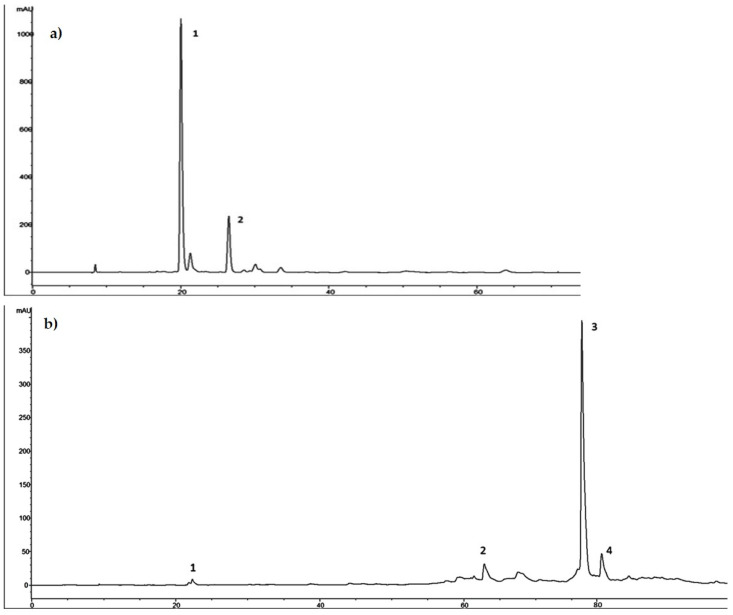
(**a**) Chromatographic profile of hydroxytyrosol-enriched extract (HTyrE) at 280 nm. 1. HTyr; 2. Tyrosol. (**b**) Chromatographic profile of oleuropein-enriched extract (OleE) at 280 nm. 1. HTyr; 2. verbascoside; 3. oleuropein; 4. secoiridoid derivative.

As showed in the chromatographic profile recorded at 280 nm and reported in Figure 2a, HTyr proved to be the main component of HTyrE with 115.24 mg/g (77%, *w*/*w*) and tyrosol was the secondary component with 34.50 ± 1.04 mg/g (23% *w*/*w*).

Ole was the main component of OleE with 341.47 ± 13.66 mg/g (89%, *w*/*w*); HTyr, verbascoside and a secoiridoid derivative were the secondary components with 1.2 ± 0.04 mg/g, 2.53 ± 0.08 mg/g and 38.12 ± 1.22 mg/g, respectively, for a total of 11% *w*/*w* (Figure 2b).

**Figure 3:**
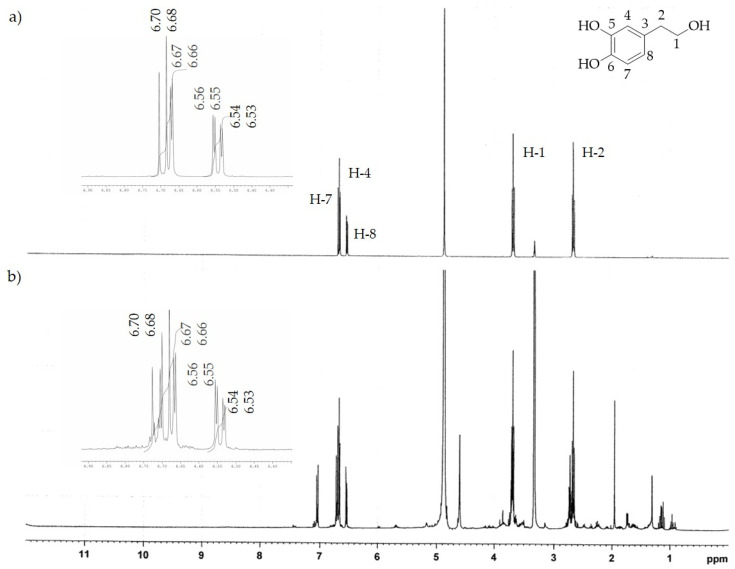
^1^H-NMR spectrum of (**a**) HTyr; (**b**) HTyrE.

**Figure 4:**
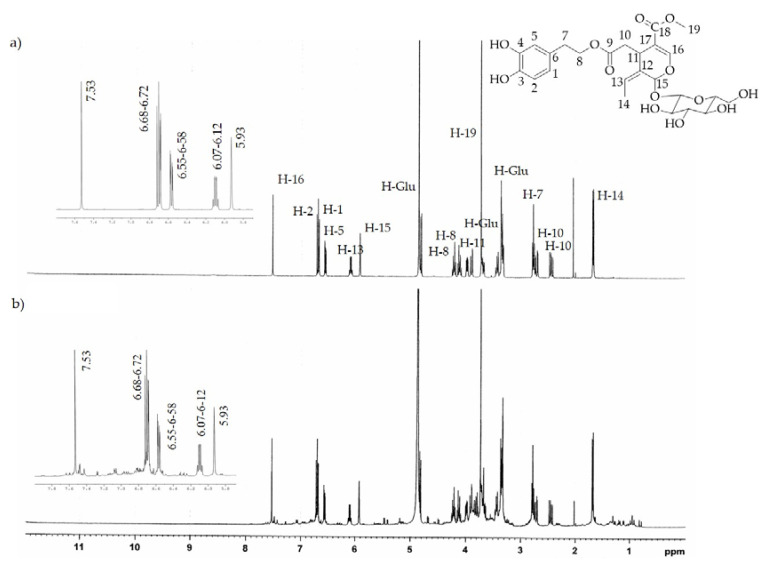
^1^H-NMR spectrum of (**a**) pure Ole; (**b**) Ole enriched extract (OleE).

The ^1^H-NMR analysis of both extracts confirmed the chemical compositions determined by HPLC-DAD. In fact, the characteristic absorption signals of pure HTyr and Ole [32,33] found by ^1^H-NMR spectrum analysis (see Figure 3a and Figure 4a) were predominantly present in the HTyrE and OleE spectra (see Figure 3b and Figure 4b) clearly proving that HTyr and Ole are indeed the main components of HTyrE and OleE, respectively.

Similar conclusions can be drawn comparing ^13^C-NMR spectra of HTyrE and OleE to those of pure HTyr and Ole (data not showed).

**Figure 5:**
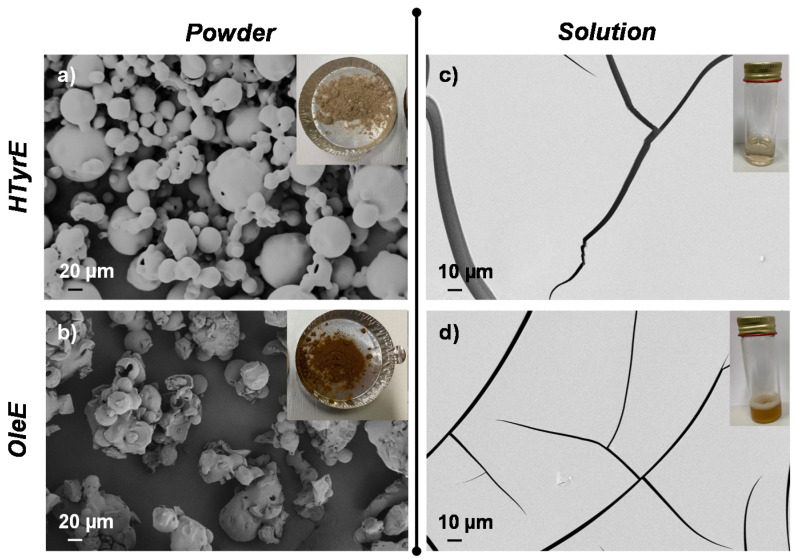
Visual aspect and field emission scanning electron microscopy (FESEM) images of (**a**) HTyrE and (**b**) OleE powder; (**c**,**d**) after dispersion in water.

Visual appearance and morphological analysis of HTyrE and OleE as powder or in solution are shown in Figure 5. HTyrE powder showed a light beige color (Figure 5a). Field emission scanning electron microscopy (FESEM) images evaluation shows that HTyrE pristine powder was characterized by spherical shape in accordance with literature (Figure 5a) [34]. FESEM investigation also showed that active powder dimensions ranged in two different micrometer populations distributed between 20–40 µm and 50–80 µm. Visual observation and FESEM analysis of HTyrE in aqueous solution are reported in Figure 5c. HTyrE solution resulted totally transparent (insert Figure 5c) maintaining the same color previously detected for the powder, the transparency and solubility were guaranteed by the use of water as solvent. Morphological investigation of the HTyrE solution highlighted the efficiency of the selected procedure adopted to obtain the AI dispersion by modifying the dimension of the initial crystals. In water solution, the AI completely lost the initial shape, in fact during the evaporation process the different drops of HTyrE solution created a film, a behavior ascribable to its high hydrophilic nature [13,35].

Similar analysis and observations were also obtained for both OleE powder and solution. OleE powder presented a brown-yellowish color (see insert Figure 5b) characterized by microparticles with circular/spherical shape (FESEM image, Figure 5b) [36]. However, FESEM investigation showed that the powder distributed in two different micrometer dimension populations ranged in this case between 10–30 µm and 40–80 µm. In Figure 5d is shown the visual observation and FESEM image of OleE in water dispersion. The OleE solution was partially transparent (insert Figure 5d), the aqueous solution maintained the same color observed for the powder. Additionally, in the case of the OleE solution, the morphological investigation highlighted the efficiency of the selected water solvent procedure that certainly modified the dimension of the initial crystals. In fact, as observed for HTyrE, the AI lost completely the initial shape in water solution and during the evaporation process the different drops of OleE solution created a film, behavior related to the high hydrophilic nature of the pristine ingredient.

**Figure 6:**
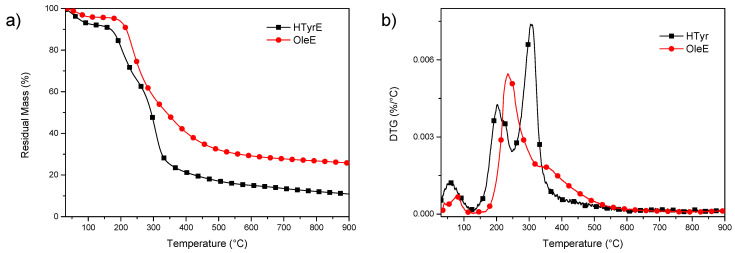
TG (residual mass) (**a**) and DTG (derivative weight loss) (**b**) curves for HTyrE and OleE.

Thermal stability of OleE and HTyrE under inert atmosphere was analyzed by thermogravimetric analysis (TGA) (Figure 6a,b). The experimental results showed that both extracts have limited thermal resistance, with initial mass loss due to water evaporation up to 130 °C. Subsequently, additional mass loss occurred, with a significant increase in weight loss rate up to 300 °C. In particular, a visible increase of the degradation rate was observed in the case of HTyrE, with the first DTG (derivative weight loss) peak occurring at 203 °C and the main peak, centered at 305 °C, due to the decomposition of more stable compounds [12,37,38]. Mass loss was 73% in the interval between temperatures 200 and 500 °C, which suggested the cracking of –C–C–, –C=C–, and –OH bonds to further form CO_2_, H_2_O, and carbonaceous material [39]. In the case of OleE, the maximum degradation peak was shifted to 235 °C, with a shoulder at 352 °C; the increased thermal stability compared to HTyrE can be related to the more complex chemical structure of OleE, which is able to justify the delayed thermal degradation [40,41]. The higher residual mass at the end of the test for OleE (25.6%) when compared with HTyrE (10.9%) is also ascribable to the higher tendency of OleE to charrification.

### 2.2. Characterization of PVA-Based Films

#### 2.2.1. Morphological, Transparency and Color Properties

**Figure 7:**
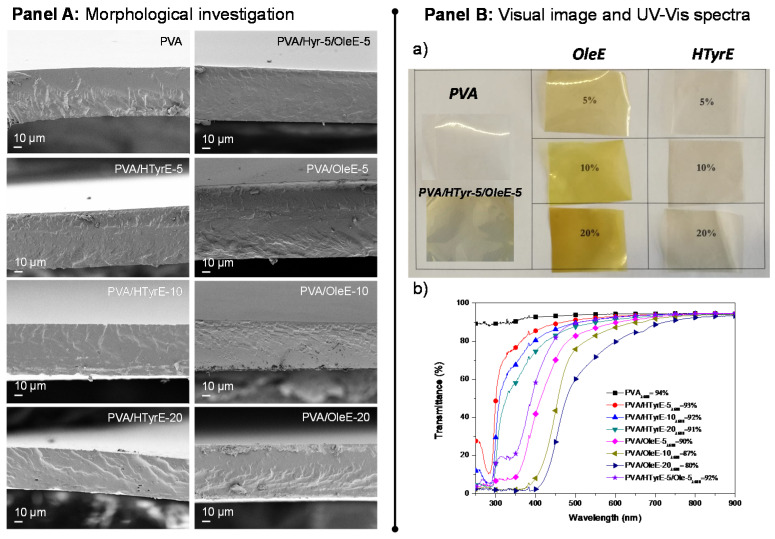
Panel A: FESEM images of fractured surfaces of poly (vinyl alcohol) (PVA), PVA/HTyrE and PVA/OleE films. Panel B: (**a**) Visual image and (**b**) UV-Vis analysis of PVA, PVA/HTyrE and PVA/OleE films.

The morphology of the cross-section of PVA films combined with HTyrE and OleE at different percentages (5, 10 and 20 %wt) was investigated via FESEM (Figure 7, Panel A). A homogeneous and smooth internal fractured surface was found for neat PVA film, as commonly [42] detected for well processed thermoplastic semi-crystalline polymers and copolymers [38]. The addition of HTyrE and OleE in cast based formulations did not negatively affected the fractured surfaces, no evident alterations were observed in the corresponding PVA-based films in terms of homogeneity and uniformity for both extracts at the different concentrations tested. This positive behavior was ascribable to the good dispersion of each extract in the PVA matrix processed by solvent casting, indeed also favored by the high solubility in water [13,35].

Visual aspect and transparency of PVA and PVA-based films were also analyzed. Figure 7, Panel B Sections (a) and (b) show visual observations and UV-Vis characterizations of PVA, PVA/HTyrE and PVA/OleE films respectively. The image of PVA-based films displays the color of the different produced samples and the homogeneity of dispersion of HTyrE and OleE.

The results from UV-Vis analysis, performed for all films, confirmed the transparent nature of PVA film (transmittance of 94% at a wavelength of 600 nm), this high value was only slightly affected by the addition of HTyrE at different concentrations (transmittance at 600 nm: PVA/HTyrE-5 = 93%, PVA/HTyrE-10 = 92% and PVA/HTyrE-20 = 91%) [12,13]; on the other hand, the presence of OleE affected the transparency of the resulting film more remarkably (Figure 7 Panel B, b). The transmittance trend was kept stable up to wavelength values of 450 and 550 nm for PVA/HTyrE and PVA/OleE films, respectively. This behavior highlights the possibility to generate transparent systems in the wavelength range of 600–900 nm. Furthermore, the transparency behavior for PVA/HTyrE films rapidly decreased from 450 nm to lower wavelength values. PVA/HTyrE films showed a visible absorption band centered at 280 nm as a consequence of the presence of HTyrE [13].

The novel films exhibited a versatile behavior according to the wavelength range, since the addition of different extracts clearly affected the absorption of UV light (from 250–500 nm). Precisely, the addition of OleE reduced drastically the transmittance of UVA (315–400 nm) and UVB (280–315 nm), while HTyrE limited the transparency in the UVA region and increases the protection in the UVB region (lower transmittance in UVB region respect to UVA wavelengths). In this respect, the development of PVA/HTyrE-5/OleE-5 combined the dual and positive behavior of each enriched extract. This behavior can act positively guaranteeing an increase in shelf life to light sensitive food, since clearly the extracts hindered the passage of light radiation of specific wavelengths that could damage the packaged products [13,43].

The results obtained for the color analysis of PVA based films are summarized in Table 1. The colorimetric and gloss analysis were performed to determine the effect of HTyrE and OleE on the optical and aesthetic appearance of the films.

Neat PVA film is characterized by high lightness value (L* = 99.16 ± 0.04) respect to the other types of films. The measured value indicated high transparency of the matrix and a slight reduction of transparency when HTyrE or OleE was added into PVA, in accordance with the results of UV-Vis characterization (Figure 7 Panel B, b). The b* values were positive for all formulations and indicated the chromatic tendency of these films to take on shades of yellow, particularly the OleE based ones. The addition of the extracts determined a colorimetric deviation from lighter yellow (desert sand) to darker yellow/brown using HTyrE or OleE, respectively. These variations were magnified by increasing the concentration of both extracts (Table 1) and using OleE (b* PVA = 0.13 ± 0.02, b* PVA/20HTyrE = 9.17 ± 0.06 and b* PVA/20OleE = 55.76 ± 0.13). The increase in concentration significantly contributed in modifying the ΔE* values. The highest ΔE* value for PVA films has been registered for PVA/OleE-20 film (ΔE* = 60.14 ± 0.37). The PVA/HTyrE-5/OleE-5 showed similar color parameters of PVA/OleE-5 clearly revealing that OleE greatly influenced the final color of the polymeric system. The presence of the extracts influenced the aesthetic quality of PVA-based films; this characteristic was expressed through the gloss value that was reduced as the concentration of the extracts increased [8,43].

#### 2.2.2. Thermal and Mechanical Behavior

Thermogravimetric analysis (TGA) and differential scanning calorimetry (DSC) under nitrogen atmosphere have been performed on the different PVA based formulations to evaluate the effect of the extracts on films properties.

**Figure 8:**
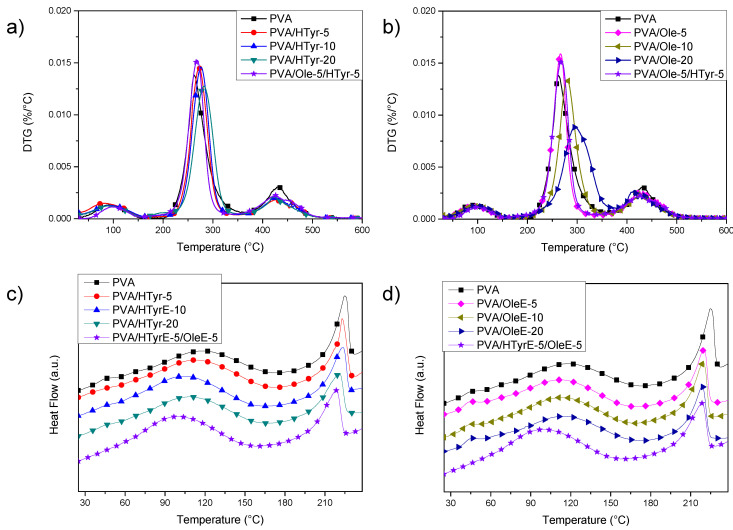
DTG curves (**a**,**b**) and first differential scanning calorimetry (DSC) heating scans (**c**,**d**) for neat PVA, PVA/HTyrE and PVA/OleE films.

TGA results (Figure 8a,b) showed an initial weight loss up to 10% for neat PVA when the temperature reached 140 °C. This loss is due to the evaporation of the adsorbed water. Subsequently, two main peaks were found, with a maximum at 262 °C and 429 °C, corresponding to two distinct decomposition steps. The first temperature can be assigned to the side chain of the PVA, while the second temperature can be related to a decomposition of the main chain of PVA [44]. Adding polyphenols to PVA positively affected the thermal stability of the films, since the progressive addition of natural stabilizers slowed down polymer decomposition. According to the gathered data, PVA with OleE was the most thermally stable system, with a shift from 262 °C to 296 °C for the main peak measured in the case of PVA/OleE-20. In addition, HTyrE improved the thermal stability of polyvinyl alcohol, even if at a limited extent: in this case, analysis of the DTG curves revealed a shift of the second peak from 262 to 282 °C for PVA/HTyrE-20, that in fact reached the maximum level with the highest content of the additive [45]. The temperature for the second main peak in the case of hybrid PVA/ HTyrE-5/Ole-5 system was found at 269 °C, between the two respective maximum degradation rates (measured at 266 °C for PVA/OleE-5 and 272 °C for PVA/HTyrE-5.

In accordance with the results of TGA characterization (Figure 6), where we found thermal degradation of OleE and HTyrE beyond 130 °C [46], and by considering that PVA needs to be heated up to 240 °C to clearly identify its thermal behavior, we limited DSC analysis to a first heating scan. In fact, heating to 240 °C of PVA films loaded with OleE and HTyrE will induce thermal degradation of the extracts, altering the thermal response of the PVA matrix. The analysis of the curves (Figure 8c,d) showed the presence of a transition, visible at 40 °C, related to wet fraction of PVA [47]. In fact water acts as a plasticizer in wet samples, so the expected increase in glass transition temperature to about 70 °C in a second heating scan can be explained by the evaporation of water during the first scan reported as an endothermic peak in Figure 8c,d. On the other hand, a shift towards lower temperatures of PVA melting peak was noted. A melting peak for neat PVA was registered at 225 °C, while the same event appeared at 218 °C and 220 °C, respectively for PVA_20OLE and PVA_20HTyr, without any substantial variation in the case of PVA/HTyrE-5/OleE-5 (melting peak at 219 °C). The same behavior was observed for poly(lactic acid) (PLA) based films [48].

Results of tensile for PVA films with the various concentrations of HTyrE or OleE are presented in Table 2. Mean values of stress and strain at break for neat PVA film were equal to 39 MPa and 101%, respectively. In the case of films containing HTyrE up to 10%wt, the additive was effective in enhancing tensile strength, while in terms of the deformation at break, a lower limit was detected for the concentration of 5%wt. In particular, with the increase of HTyrE content we observed a change to 140 and 105% for the ε_b_ and from 56 to 59 MPa for the σ_b_, respectively. Beyond this level, the content of the extract was too high and an antiplasticization effect was registered (the value for the deformation at break was lower than neat PVA, with a parallel increase in stiffness) [49,50]. A similar behavior was found for OleE containing films, which indeed proved to be more resistant and less ductile of the corresponding HTyr containing films (a change to 166 and 163% for the ε_b_ and from 53 to 57 MPa for the σ_b_, respectively, with increasing loading of OleE): comparing the free hydroxyl groups on HTyrE and OleE, it is expected that the different abundance of these groups limited the formation of strong hydrogen bonds in the case of HTyrE, while they can be predicted in the case of OleE. Moreover the enhanced of thermal stability of the extract and of the corresponding PVA/OleE films observed by TGA supports this assumption [51].

#### 2.2.3. Total Phenolic Content (TPC) Analysis

Total phenolic content (TPC) analysis and antioxidant activity were measured as an indirect way to evaluate the release properties and the antiradical activity of the produced films, and therefore to select the better concentration of HTyrE and OleE to be incorporated in the PVA films. Indeed, an important property of the developed formulations is the antioxidant activity induced by the AI, which is directly linked with the phenolic content; therefore, the release properties of active components and their antioxidant activity after migration was analyzed.

For this purpose, different formulations (PVA/HTyrE-5, PVA/HTyrE-10, PVA/HTyrE-20, PVA/OleE-5, PVA/OleE-10 and PVA/OleE-20) have been prepared and tested for TPC and antioxidant activity through the Folin–Ciocâlteau and the 2,2-diphenyl-1-picryl-hydrazyl-hydrate (DPPH) assays, respectively.

**Figure 9:**
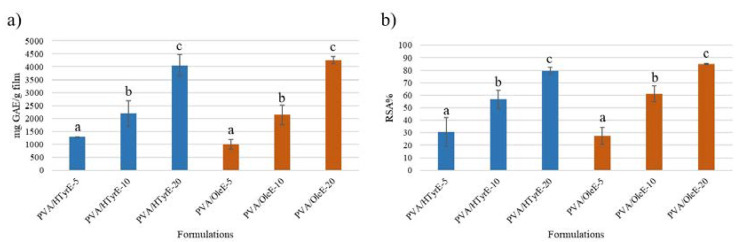
(**a**) Total phenolic content (TPC) and (**b**) antioxidant activity of PVA/HTyrE-5, PVA/HTyrE-10, PVA/HTyrE-20, PVA/OleE-5, PVA/OleE-10 and PVA/OleE-20 formulations determined by Folin–Ciocalteu method and 2,2-diphenyl-1-picryl-hydrazyl-hydrate (DPPH) assay respectively. Results are expressed as mg of gallic acid equivalent (GAE)/g of film for TPC and as radical scavenging activity (*RSA*%) for DPPH assay. Significant differences for *p* ≤ 0.05 were evidenced performing Tukey HSD test and are indicated with different letters.

The results, depicted in Figure 9a, showed a corresponding increase in TPC value in relation to the phenolic concentration of the formulations. A similar trend was observed for the antioxidant activity determined by DPPH assay on the solutions obtained from each formulation [43]. Indeed, as showed in Figure 9b, all formulations exhibited an antioxidant activity value expressed as radical scavenging activity (*RSA*%) value in direct relation to the phenolic concentration present in the formulations.

The 10% *w*/*w* concentration of HTyrE and OleE used for casting the PVA films was chosen on the basis of the transparency behavior as well as balancing morphological features, optical properties and antioxidant performances of the resulting materials. In fact the presence of a higher content of the extract (20% *w*/*w*, PVA/HTyrE-20 and PVA/OleE-20) radically reduce the transparency in the visible spectrum around wavelengths (λ) of 400–700 nm (Figure 7 Panel B, b).

In addition, a blend of HTyrE (5% *w*/*w*) and OleE (5% *w*/*w*) was selected to verify a possible synergic effect of both extracts on the properties of the corresponding formulations.

#### 2.2.4. Specific Migration, and Antioxidant Activity

**Figure 10:**
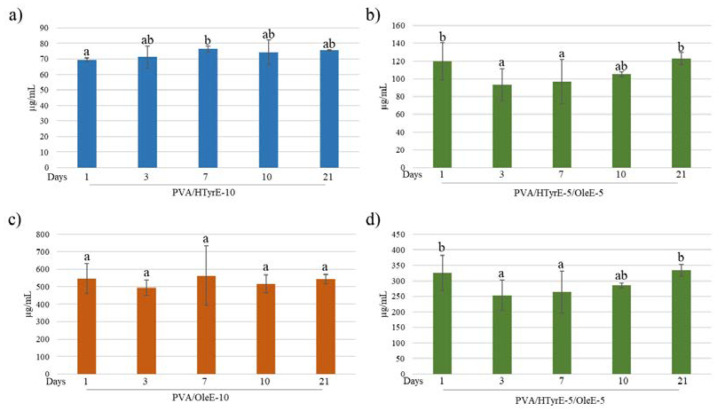
(**a**,**b**) HTyr and (**c**,**d**) Ole released (µg/mL) from each formulation (PVA/HTyrE-10, PVA/OleE-10 and PVA/HTyrE-5/OleE-5) into the simulant solution at different contact times. Analysis have been carried out in a food simulant (ethanol 10% *v*/*v*) for 21 days. Significant differences for *p* ≤ 0.05 were evidenced performing Tukey HSD test and are indicated with different letters.

The antioxidant activity of PVA/HTyrE-10, PVA/OleE-10 and PVA/HTyr-5/OleE-5 was evaluated in a food simulant to test their effectiveness as active packages. Release tests were performed according to the European Standard EN 13130-2005 [52] and European Commission Regulation 10/2011 [53]. Each formulation was analyzed in the proper water-based foods simulant solution (ethanol 10% *v*/*v*) for 21 days. All samples were analyzed at 1, 3, 7, 10 and 21 days quantifying HTyr and Ole released via UV-Vis spectrophotometry. As shown in Figure 10, in all tested formulations, concentration of HTyr and Ole did not sensibly increase during the 21 days of the test.

**Figure 11:**
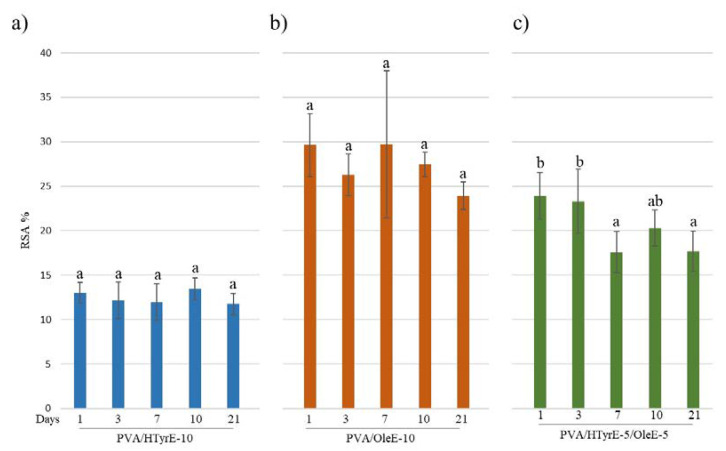
Radical scavenging activity (*RSA*%) of food simulant solutions after migration tests from PVA/HTyrE-10 (**a**), PVA/OleE-10 (**b**) and PVA/HTyrE-5/OleE-5 (**c**) formulations. Significant differences for *p* ≤ 0.05 were evidenced performing Tukey HSD test and are indicated with different letters.

The antioxidant activity was evaluated through the DPPH assay as well performed on the food simulant solutions at different days (1, 3, 7, 10 and 21 days). As shown in Figure 11, a remarkable antioxidant activity was observed for all formulations tested over time. In line with the experimental data concerning the specific migration, antioxidant activity did not sensibly increase during the 21 days of the test. These results prove an uncontrolled quick release and are indeed in total accordance with what has been observed for PVA films incorporating pure active ingredients such as HTyr, Gallic acid and quercetin into PVA [10,12].

As depicted, all formulations showed antioxidant activity in the following order: PVA/OleE-10, PVA/HTyrE-5/OleE-5, and PVA/HTyrE-10. These results were consistent with the greater concentration of Ole into OleE compared to that of HTyr into HTyrE (90% and 77%, respectively).

## 3. Materials and Methods

### 3.1. Materials

*Olea europaea* L. wastes and by-product (free-oil olive pulp and leaves) were obtained from Leccino cultivar (Vinci, Florence, Italy). Leaves were collected in April 2019 during the pruning of olive trees; free-oil olive pulp in October 2019 after oil extraction. Poly (vinyl alcohol) (average MW 85–124 kg mol^−1^, 99% hydrolyzed) was purchased from Sigma-Aldrich^®^ (Milan, Italy). Chemicals and HPLC grade solvents were purchased from Sigma-Aldrich (Milan, Italy). Tyrosol and oleuropein used as standards were supplied by Extrasynthèse (Genay, France). Hydroxytyrosol was synthesized according to an optimized and patented procedure [32].

### 3.2. Hydroxytyrosol-Enriched Extract (HTyrE) and Oleuropein-Enriched Extract (OleE) Preparation

HTyrE and OleE were prepared from free-oil olive pulp and leaves respectively using an industrial process for recovery and concentration, as already described [54]. By applying a spray drying process at the end of the extraction, a powder for each sample was obtained. Flash and preparative chromatography were carried out by a HPLC-PURE C-850 (Büchi Italia Srl, Cornaredo, Milan, Italy) equipped with a Sepacore pump C-605/control module C-615-T-valve, backpressure regulators from Pure sampling pump-column FlashPure Ecoflex C18 cartridges. For each run, 600 mg of the sample were introduced in the chromatography system with a syringe and purified. The eluents were water (Eluent A) and ethanol (Eluent B). A five-step linear solvent gradient was used starting from 20% of Solvent B for two minutes, for 3 min at 70% of Solvent B and for 14 min at 100% of Solvent B at a flow rate of 30 mL/min. The UV wavelengths were 240 nm, 285 nm, 330 nm and 310 nm (UV threshold: 0.05 AU). The fractions obtained after the chromatographic process were concentrated under vacuum, at 25 °C using a Rotavapor B-490 (Büchi Italia Srl, Cornaredo, Milan, Italy); the resulting samples were then concentrated in water and freeze-dried using the Lyovac GT2 freeze dryer (Leybold-Heraeus, Cologno Monzese, Milan, Italy) at T < −30 °C and P = 1 bar.

### 3.3. HTyrE and OleE Characterization

#### 3.3.1. High Performance Liquid Chromatography/Diode Array Detector (HPLC-DAD) Analysis

Qualitative and quantitative content of HTyrE and OleE were determined using a HP 1260 liquid chromatograph (Agilent Technologies, Palo Alto, CA, USA) equipped with an analytical column Lichrosorb RP18 250 × 4.60 mm i.d, 5 μm (Merck Darmstadt, Germany). The eluents were water adjusted to pH = 3.2 with formic acid (Solvent A) and acetonitrile (Solvent B). A four-step linear solvent gradient was used starting from 100% of Solvent A up to 100% of Solvent B, for 88 min at a flow rate of 0.8 mL × min^−1^. Polyphenols found in the extracts were identified by comparing retention times and UV/Vis spectra with those of the authentic standards. Each compound was quantified at the selected wavelength (240, 280, 330, and 350 nm) using a five-point regression curve [55].

#### 3.3.2. Nuclear Magnetic Resonance (NMR) Analysis

HTyr or Ole (20 mg) and HTyrE or OleaE (30 mg) were solubilized in methanol-d4 (0.5 mL). ^1^H-NMR and ^13^C-NMR spectra were recorded using the 400 MHz Nuclear Magnetic Resonance Spectrometer Avance III (Bruker Italia, Milan, Italy). Chemical shifts were expressed in parts per million (δ scale) and referred to the residual protons of the solvent.

#### 3.3.3. Morphological Characterization

The morphological investigation of HTyrE and OleE in powder state and after dissolution in deionized water was performed by field emission scanning electron microscopy (FESEM, Supra 25-Zeiss, Oberkochen, Germany). Powders were deposited on conductive adhesive, gold sputtered and analyzed, a similar procedure was also adopted for the AI in water solution. Few drops of the AI suspensions were deposited onto a substrate of silicon, dried at room temperature (RT) and visualized after gold sputtering. A magnetic stirring at RT for 1 h was applied in order to obtain a uniform dispersion of the Ais in the solvent.

#### 3.3.4. Thermal Characterization

Thermogravimetric measurements (TGA) of HTyrE and OleE powders were performed under inert atmosphere (nitrogen flow) by using a Seiko Exstar 6300 (Tokyo, Japan). Heating scans were performed from 30 to 900 °C at 10 °C min^−1^ and three repetitions of the test were considered.

### 3.4. Preparation of PVA-Based Formulations

PVA-based films were prepared by solvent casting technique. The content of HTyrE and OleE incorporated were 5, 10 and 20%wt. in accordance with available literature [10,40] and then fixed at 10%wt for each formulation [10,40]. Initially, PVA (0.5 g) was dissolved in distilled water (10 mL) under magnetic stirring for 2 h at 80 °C. The polymeric solution was kept under magnetic stirring to reach RT and then cast. PVA-based films listed in Table 3 were obtained mixing, under magnetic stirring, the polymeric solution with a specific amount of HTyrE and/or OleE. Each extract was previously dispersed in distilled water (0.1 g of extract in 10 mL of distilled water) for 1 h at RT.

Homogeneous HTyrE or OleE dispersions in aqueous solution were obtained applying magnetic stirring and sonication, both at RT for 1 h (Figure 10).

**Figure 12:**
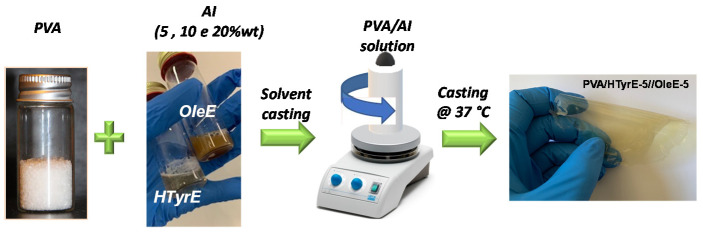
Procedure for the preparation of novel PVA formulations.

The polymeric solutions were cast in a Teflon^®^ substrate and the evaporated at RT. Figure 12 shows a schematic representation of the process step of PVA films. PVA films with thicknesses between 50 and 80 µm were equilibrated for 1 week at 53% RH in desiccators before characterizations. The RH conditions during storage were guaranteed using magnesium nitrate-6-hydrate oversaturated solution [12,56].

### 3.5. Characterization of PVA-Based Formulations

#### 3.5.1. Morphological and Color Analysis

Field emission scanning electron microscopy (FESEM, Supra 25-Zeiss, Oberkochen, Germany) was used to investigate the effect of both HTyrE and/or OleE on the fracture surface of PVA based films. The surfaces were gold sputtered in order to offer electric conductivity and the samples were observed using an accelerating voltage of 2.5 kV.

The color parameters of PVA films were determined by means of a spectrophotometer (CM-2300d Konica Minolta, Tokyo, Japan). Data were acquired by using the SCI 10/D65 method whereas CIELAB color variables, as defined by the Commission Internationale de l′Éclairage (CIE 1995), were utilized. The different produced formulations were positioned on a white standard plate and L*, a*, and b* parameters were analyzed. L* value ranges from 0 (black) to 100 (white); a* value ranges from −60 (green) to 60 (red); and b* value ranges from −60 (blue) to 60 (yellow). Samples were evaluated in triplicate, and three measurements were taken at random locations on each of the studied films. The total color difference ΔE* and gloss parameters were calculated as indicated in following equation:

(1) $$\Delta E^{*}\;*=\sqrt{{\left(\Delta L^{*}\right)}^{2}+{\left(\Delta a^{*}\right)}^{2}+{\left(\Delta b^{*}\right)}^{2}}$$

#### 3.5.2. Thermal Characterization

Thermal characterization of PVA based formulations was determined by thermogravimetric measurements (TGA) and by differential scanning calorimetry (DSC). Thermogravimetric tests were performed by using a Seiko Exstar 6300 (Tokyo, Japan). Heating scans from 30 to 600 °C at 10 °C min^−1^ under nitrogen flow were performed for each sample.

Differential scanning calorimetric measurements were performed on a TA Instruments DSC Q200 (TA Instruments Inc., New Castle, DE, USA) under nitrogen flow in the range from −25 to 240 °C at 10 °C min^−1^, carrying out only a fist scan.

#### 3.5.3. Mechanical Characterization

The mechanical properties of PVA based formulations were evaluated by tensile tests by using rectangular probes on the basis of UNI ISO 527 standard with a load cell of 500 N and a crosshead speed of 5mm min^−1^. The tensile strength (*σB*) and elongation at break (*εB*) were calculated from the stress-strain curves. The analysis was carried out at RT and five samples for each material were tested. Samples were tested after being stored for one week at RT in a desiccator containing silica salts.

#### 3.5.4. Total Phenolic Content

TPC of PVA/HTyrE-5, PVA/HTyrE-10, PVA/HTyrE-20, PVA/OleE-5, PVA/OleE-10 and PVA/OleE-20 formulations was measured through the Folin–Ciocâlteu method [57]. Briefly, each PVA system (0.1 g) was placed in methanol (2 mL) for 24 h at RT [35]. Then, 100 µL of samples or standard, 2 mL of H_2_O and 200 µL of Folin–Ciocâlteau reagent were mixed and incubated at RT for 3 min. Next, 1 mL of Na_2_CO_3_ (20/80 *p*/*v*) was added and the solutions were placed in the dark for 1 h. Finally, absorbance was measured at 765 nm using a UV/VIS spectrophotometer (UV-2600-Shimadzu, Kyoto, Japan). TPC values were quantified using gallic acid (GA) as standard (300–590 mg/L) and expressed as mg of Gallic acid equivalent (GAE)/g of extract.

#### 3.5.5. DPPH Radical Scavenging Activity

Radical scavenging activity was determined using the DPPH assay [10]. Analyses were conducted on PVA/HTyrE-5, PVA/HTyrE-10, PVA/HTyrE-20, PVA/OleE-5, PVA/OleE-10 and PVA/OleE-20 methanolic solutions and on food simulant solutions from PVA/HTyrE-10, PVA/OleE-10 and PVA/HTy-5/OleE-5 at every contact time (1, 3, 7, 10, and 21 days). All solutions (1 mL) were mixed with 1 mL of 2,2-diphenyl-1-picryl-hydrazyl-hydrate (DPPH) in methanol (50 mg/L) and incubated at RT in the dark for 60 min. The absorbance was measured at 517 nm using a UV/VIS spectrophotometer (UV-2600-Shimadzu, Kyoto, Japan). DPPH radical scavenging activity (*RSA*) was calculated according to the following equation:

$$RSA\;\left(\%\right)=\frac{A_{control}-A_{sample}}{A_{control}}\times 100$$

where *A_sample_* (R.B.).

#### 3.5.6. Release Studies in Food Simulant

Specific migration tests were performed into ethanol/water 10% (*v*/*v*) solution as food simulant, according to European Standard EN 13130-2005 [52] and European Commission Regulation 10/2011 [53]. Double-sided, total immersion migration tests were carried out with 12 cm^2^ of films and 20 mL of simulant (area-to-volume ratio around 6 dm^2^ L^−1^) in triplicate at 40 °C in an oven. Samples were taken at 1, 3, 7, 10, 21 days in triplicate; a blank test for the simulant was also included. After the migration tests, each solution was recovered and stored at −4 °C before analysis.

HTyrE and OleE released by each PVA-based formulation in food simulant (10% (*v*/*v*) ethanol water solution) for every contact time (1, 3, 7, 10, and 21 days) were analyzed using a UV/VIS spectrophotometer (UV-2600-Shimadzu, Kyoto, Japan). Absorbance values were measured at 280 nm. Standard curves for HTyr (8–60 µg/mL) and Ole (25–110 µg/mL) were used. Samples were analyzed in triplicate and results are reported as the average ± SD and expressed as µg of antioxidant per mL of food simulant.

### 3.6. Statistics

Data were expressed as averages  ±  SD of three replicate determinations. Statistical differences were calculated using one-way analysis of variance (ANOVA), followed by Tukey’s honest significant difference (HSD) post hoc test.

## 4. Conclusions

Novel films were designed and produced incorporating HTyrE and OleE as active ingredients into PVA by solvent casting method. Both extracts were obtained from olive oil wastes and by-products (free-oil olive pulp and leaves, respectively) through a sustainable processes based on membrane technologies and finely characterized by analytical advanced techniques as HPLC-DAD and NMR. The resulting films (PVA/HTyrE-10, PVA/OleE-10, and PVA/HTyrE-5/OleE-5) were characterized investigating the morphological behavior, optical and antioxidant properties. All formulations showed good performances and the best antioxidant effect was observed for PVA/OleE-10, followed by PVA/HTyrE-5/OleE-5 and then PVA/HTyr-10. Based on their properties, these films seem promising as active packages for the preservation of oxidative-sensitive water based food products. A relevant environmental aspect of this work is the reuse and valorization of olive oil industry wastes and by-products according to the circular economy model.

## Figures and Tables

**Table 1:** Color coordinates of PVA/HTyrE and PVA/OleE films.

Formulations	L*	a*	b*	ΔE*	Gloss (°)
Control	99.47 ± 0.00	−0.08 ± 0.01	−0.08 ± 0.01	-	121 ± 0
PVA	99.16 ± 0.04	−0.10 ± 0.01	0.13 ± 0.02	0.37 ± 0.05	254 ± 4

PVA/HTyrE-5	97.39 ± 0.15	−0.11 ± 0.03	3.71 ± 0.22	4.33 ± 0.25	228 ± 9
PVA/HTyrE-10	95.93 ± 0.02	−0.08 ± 0.00	6.52 ± 0.11	7.49 ± 0.10	215 ± 2
PVA/HTyrE-20	94.45 ± 0.10	−0.25 ± 0.03	9.17 ± 0.06	10.53 ± 0.10	171 ± 5

PVA/OleE-5	92.41 ± 0.58	−2.98 ± 0.10	24.57 ± 1.23	25.81 ± 1.34	225 ± 3
PVA/OleE-10	87.62 ± 0.47	−2.97 ± 0.61	32.73 ± 0.12	32.73 ± 0.12	185 ± 2
PVA/OleE-20	77.50 ± 0.68	3.97 ± 0.50	55.76 ± 0.13	60.14 ± 0.37	122 ± 9

PVA/HTyrE-5/Ole-5	92.70 ± 0.89	−3.35 ± 0.12	23.59 ± 2.33	24.84 ± 2.46	222 ± 5

**Table 2:** Mechanical properties of PVA based films.

Formulations	σ_b_ (MPa)	ε_b_ (%)
PVA	39 ± 4	101 ± 15

PVA/HTyrE-5	56 ± 7	140 ± 4
PVA/HTyrE-10	59 ± 1	105 ± 14
PVA/HTyrE-20	55 ± 8	75 ± 18

PVA/OleE-5	53 ± 9	166 ± 28
PVA/OleE-10	57 ± 5	163 ± 42
PVA/OleE-20	48 ± 3	23 ± 5

PVA/HTyrE-5/Ole-5	43 ± 4	152 ± 22

**Table 3:** Material formulations of PVA and PVA based films.

Formulations	PVA	HTyrE (%wt)	OleE (%wt)
**PVA**	100	-	-

**PVA/HTyrE-5**	95	5	-
**PVA/HTyrE-10**	90	10	-
**PVA/HTyrE-20**	80	20	-

**PVA/OleE-5**	95	-	5
**PVA/OleE-10**	90	-	10
**PVA/OleE-20**	80	-	20

**PVA/HTyrE-5/OleE-5**	90	5	5

## Sample Availability

Samples of HTyrE, OleE, and PVA films are available from the authors.
